# Left ventricular assist device exchange from HeartMate II to HeartMate 3 in an Asian patient—a case report and literature review

**DOI:** 10.1186/s13019-023-02133-4

**Published:** 2023-03-07

**Authors:** Hsiao-Huang Chang, Tzu-Ting Kuo, Po-Lin Chen, Chia-Cheng Kuo, Ching-Yuan Kuo, Nai-Yuan Wu

**Affiliations:** 1 Division of Cardiovascular Surgery, Department of Surgery, Taipei Veterans General Hospital, No. 201, Sec. 2, Shipai Rd., Beitou District, Taipei, 11217 Taiwan; 2 Department of Surgery, School of Medicine, College of Medicine, Taipei Medical University, Taipei, Taiwan; 3 School of Medicine, College of Medicine, National Yang Ming Chiao Tung University, Taipei, Taiwan; 4 Institute of Biomedical Informatics, College of Life Sciences, National Yang Ming Chiao Tung University, Taipei, Taiwan

**Keywords:** Advanced heart failure, Left ventricular assist device, Pump exchange

## Abstract

**Background:**

Pump exchange surgery of left ventricular assist device (LVAD) has been demonstrated in several studies; however, information for Asian patients was limited.

**Case presentation:**

A 63-year-old man underwent a pump upgrade from HeartMate II to HeartMate 3 for driveline damage through limited left anterior thoracotomy and lower partial sternotomy. He did not experience any hemodynamic adverse events or device malfunction during postoperative follow-ups of 12 months. We also reviewed all published cases with HeartMate II exchange to HeartMate 3.

**Conclusions:**

The case demonstrated that it was safe and feasible to perform HMII LVAD exchange to HM3 through a limited approach for Asian patients.

**Supplementary Information:**

The online version contains supplementary material available at 10.1186/s13019-023-02133-4.

## Background

Because the number of heart donors is insufficient to meet the demands, the indication of left ventricular assist device (LVAD) for managing end-stage heart failure has been broadened. However, device-related complications such as infection and pump thrombosis are not uncommon [1]. While medical therapies fail to treat these complications, pump exchange surgery is required. HeartMate 3 (HM3; Abbott Laboratories, IL, USA) is a novel LVAD with a fully magnetically levitated pump rotor. Clinical trials have demonstrated that HM3 was associated with better event-free survival and fewer adverse events than HeartMate II (HMII; Abbott Laboratories, IL, USA) [2]. Pump exchange surgery allows patients to use this new-generation device with longer battery hours. Although pump exchange surgery has been reported in the previous literature [3], all pump exchanges could not be treated as the same because there are some differences in the design of each LVAD. Pump exchange to a different device is more technically challenging. Several studies have demonstrated the feasibility of pump exchange from HMII to HM3; however, information regarding Asian patients was limited [4]. Therefore, we presented an Asian patient undergoing pump exchange with an upgrade from HMII to HM3. To better understand HMII LVAD exchange to HM3, a literature review was also performed for all reported cases.

## Case presentation

**Fig. 1:**
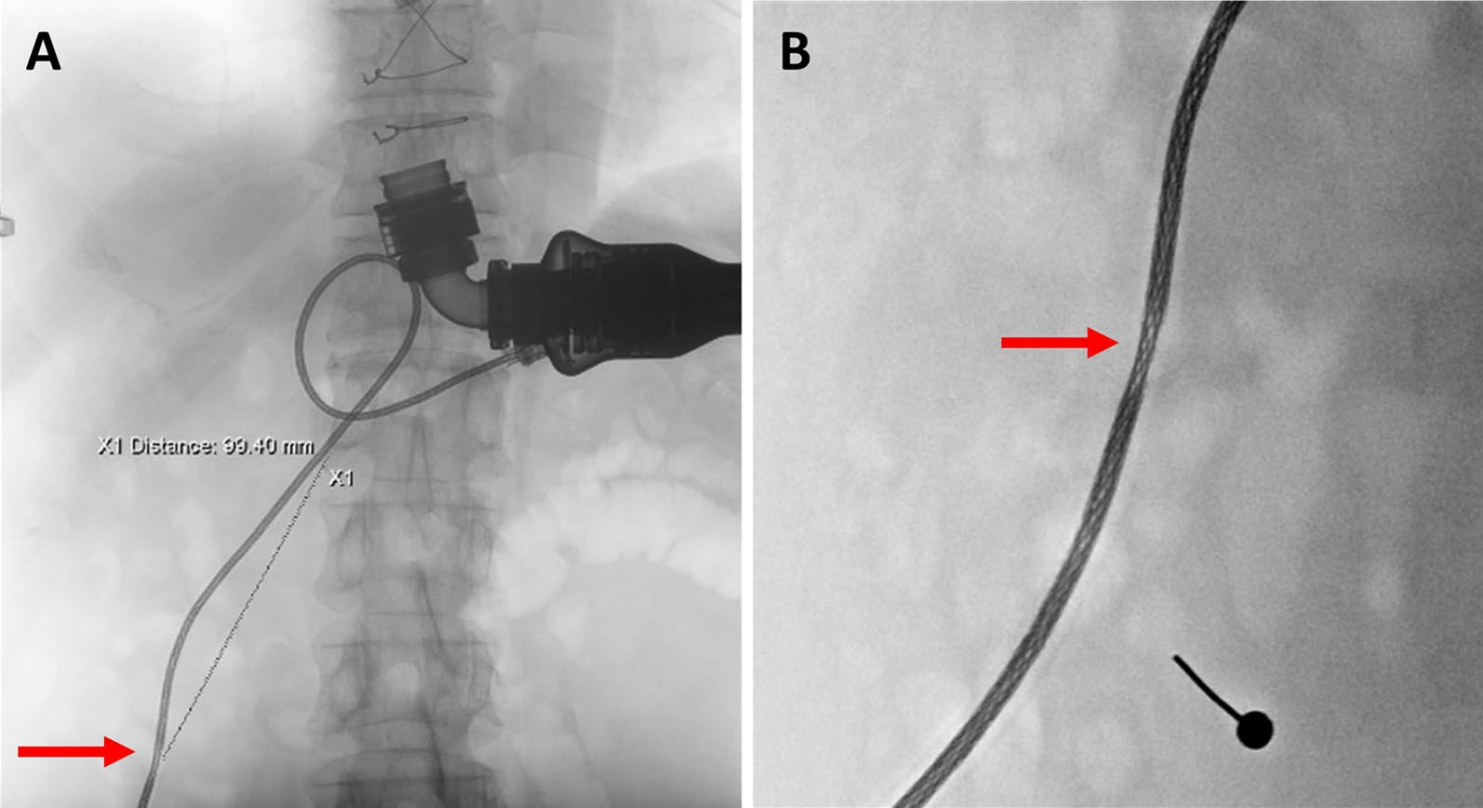
On the plain abdominal X-ray, one driveline segment (red arrows) became more radiolucent and thinner than the rest. The feature indicated the damaged part of the driveline

A 63-year-old man (body mass index: 23.1 kg/m^2^, body surface area: 1.85 m^2^) with idiopathic dilated cardiomyopathy underwent LVAD implantation with HMII as a bridge to transplantation in June 2018. In December 2020, intermittent LVAD alarms were noticed, but he did not have any discomfort. After a technical checkup, no abnormalities were found in the pump or driveline. However, intermittent pump alarms appeared again in March 2021 due to pump stoppages. The controller log file showed motor stopped 103 times in three days. On the abdominal X-ray, one driveline segment was more radiolucent and thinner than the rest, suggesting driveline damage (Fig. 1). The echocardiogram showed a left ventricular ejection fraction (LVEF) of 17%. Because the damaged driveline could not be repaired, we listed the patient on the heart transplant waitlist (status 1). However, the number of donor hearts is much lower during the COVID-19 pandemic. Due to the high risk of permanent pump stoppage, we decided to exchange the pump from HMII to HM3 instead of waiting for a heart transplant after a thorough discussion with the patient.

**Fig. 2:**
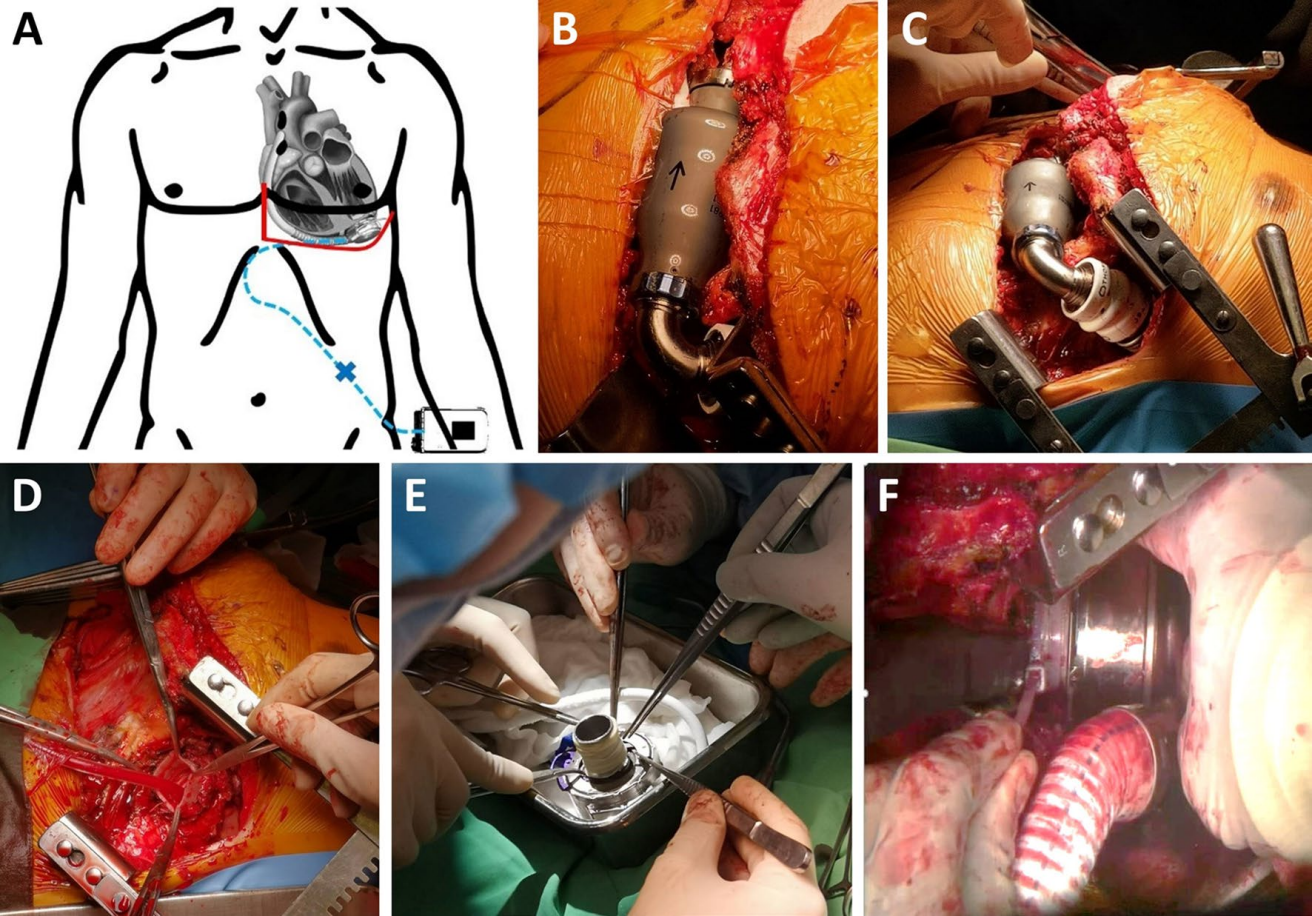
The surgical procedure of HMII pump exchange to HM3. **A** Limited left anterior thoracotomy and lower partial sternotomy were done to approach the HMII pump and the outflow graft. **B**, **C** The HMII pump was dissected out and explored. **D** After removing the tie bands, the HMII pump was removed. The inflow connector of the HMII pump was left in place. **E** Because the HMII inflow connector is larger than the HM3 inflow connector, we wrapped the inflow connector in three layers with the finger portion of the surgical glove to avoid the size mismatch problem between the HMII inflow connector and HM3 pump. **F** Then the HM3 pump was inserted into the HMII inflow connector and fixed with two tie bands (**F**)

In the pump exchange surgery, limited left anterior thoracotomy through the fifth intercostal space and lower partial sternotomy were performed to approach the HMII pump and the outflow graft (Fig. 2A). The HMII pump was dissected out and explored (Fig. 2B, 2C). The right femoral artery and vein were exposed and cannulated for cardiopulmonary bypass (CPB). After the initiation of CPB, the tie bands were removed, and then the HMII pump was removed. The inflow connector of the HMII pump was left in place (Fig. 2D). Because the HMII inflow connector is larger than the HM3 inflow connector, we wrapped the HM3 inflow connector in three layers with the finger portion of the surgical glove to avoid the size mismatch problem between the HMII and HM3 inflow connectors (Fig. 2E). Then the HM3 inflow connector was inserted into the HMII inflow connector and fixed with two tie bands (Fig. 2F). The new outflow graft was connected to the old graft by end-to-end anastomosis. The new driveline was tunneled subcutaneously to the right lower abdomen and was connected to the HM3 controller. On the old removed driveline, two breakdowns in the braided metal shield were at approximately 25 cm and 29 cm from the pump housing, respectively. After shifting CPB to HM3, the drainages were placed, and the wound was closed according to the standard procedure. The entire bypass time was 189 min.

**Fig. 3:**
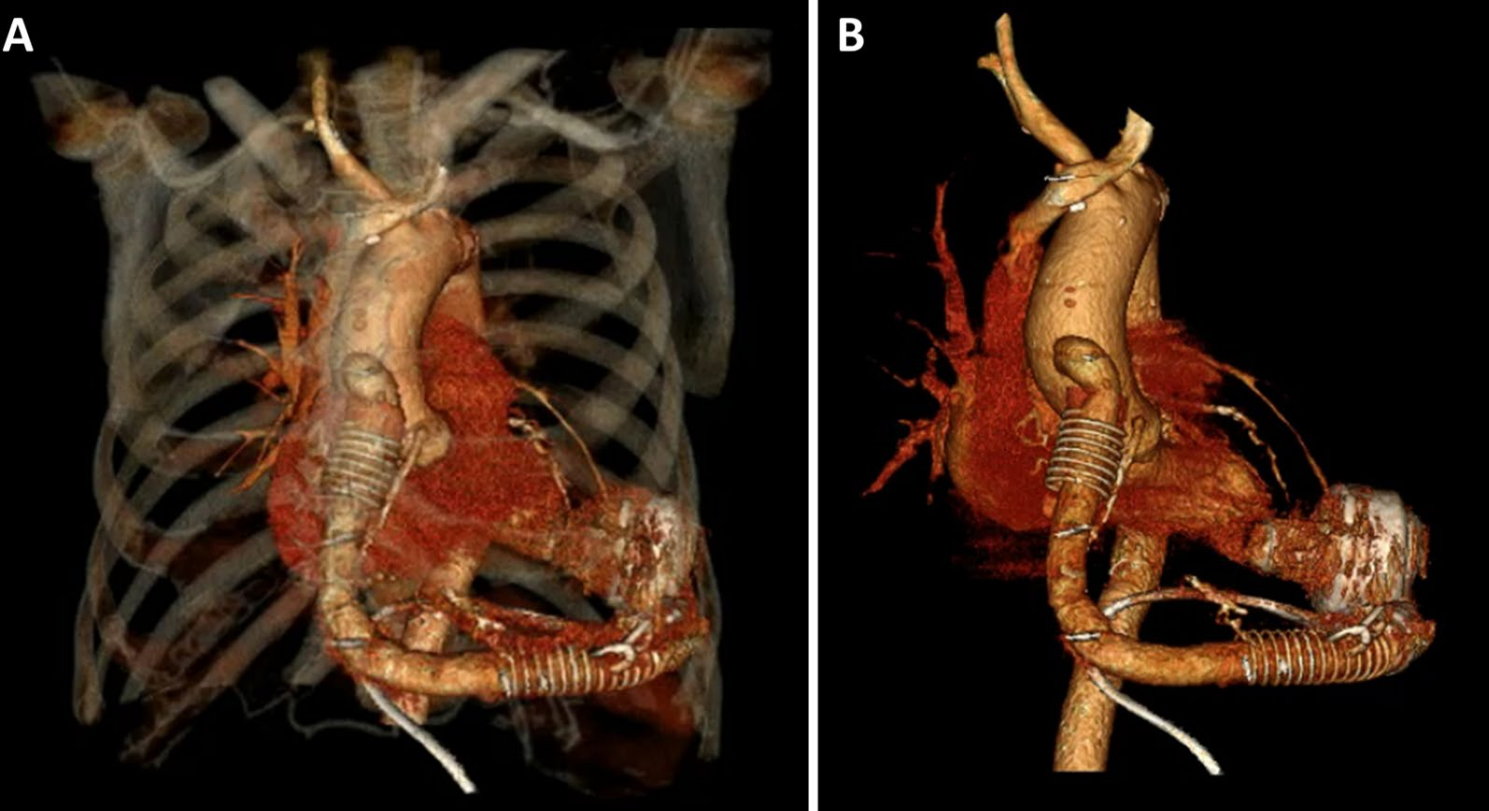
Computed tomography after pump exchange from HMII to HM3

After surgery, the patient was hemodynamically stable and had a good pump and driveline function (Fig. 3). LVEF was 46%. At postoperative week 2, he complained of cough, and cytomegalovirus pneumonia was diagnosed. Anti-viral agents with ganciclovir followed by valganciclovir were administered, and symptoms improved. At postoperative month 2, the patient received wound debridement and closure with a local flap as the previous driveline insertion site wound was poorly healed. The patient remained stable and had a good quality of life at postoperative 6- and 12-month follow-ups. The patient provided written informed consent for the publication of this report and all accompanying images.

## Literature review

The literature review methods, including search strategy, selection criteria, study selection, and data extraction, were provided in Additional file 1. Including this case report, a total of 11 studies consisting of 34 patients were reviewed (Table 1) [5–14].

**Table 1:** Summary of the included studies

1st author (year), country	N	Age	Sex	Cardiology diagnosis	Indication of LVAD	Previous LVAD	Duration of previous device	Exchange indication	Approach of pump exchange	Complications	Outcome	Duration of follow-up
Hanke [8], Hanke [7], Germany	4	56	Male	DCM	–	1	266d	Driveline infection	Lateral thoracotomy	Early: Respiratory failure	Alive	3y
		56	Male	ICM	–	1	3722d	Driveline infection	Lateral thoracotomy	Early: Respiratory failure, minor cerebral bleeding	Alive	3y
		76	Male	ICM	–	1	530d	Driveline infection	Lateral thoracotomy	–	Alive	3y
		61	Male	DCM	–	1	1475d	Pump thrombosis	Lateral thoracotomy	Early: temporary dialysis for acute renal failure Follow-up: low flow alarms, syncope	Alive	3y
Khayata [9], USA	1	34	Female	ICM	–	1	–	Pump thrombosis	Posterolateral (5t^h^ intercostal) thoracotomy, HM2 left in place, HM3 outflow to descending aorta	–	Alive	6 m
Wert [13], Germany	1	77	Male	End-stage HF	–	1	17 m	Pump prolapse	Anterolateral thoracotomy	–	Alive	–
Takeda [12], USA	9	Median 58	Male (*n* = 7), female (*n* = 2)	ICM (*n* = 3), DCM (*n* = 6)	BTT (*n* = 5), DT (*n* = 4)	1	median 608d	Pump thrombosis (*n* = 8), driveline injury (*n* = 1)	Left lateral thoracotomy toward a subcostal incision + a separate upper midline abdominal incision for outflow anastomosis	Early: Re-exploration for bleeding at HM2 pump pocket site (*n* = 1), pneumonia (*n* = 1), prolonged intubation (*n* = 2), inflow malposition at post-exchange day 7 (*n* = 1), recurrent device thrombosis (*n* = 1) Follow-up: old pump pocket infection undergoing surgical drainage and debridement (*n* = 3)	Alive (*n* = 8) death (*n* = 1)	Median 486d
Barac [6], USA	14	*Mean 56.8	*Male (*n* = 11), female (*n* = 8)	*ICM (*n* = 9), 10 Non-ICM (*n* = 10)	–	*1 (*n* = 10), 2 (*n* = 7), 3 (*n* = 2)	*Median 456d	*Malfunction (*n* = 3), infection (*n* = 2), thrombosis (*n* = 14)	Redosternotomy	*Dialysis (*n* = 1)	*Death or another replacement (*n* = 2). 90d-mortality = 100%	*Mean 221d
Radcliffe [10], USA	1	49	Male	Non-ICM	–	1	–	Pump pocket infection (MSSA)	Left subcostal thoracotomy	Prolong VRE pump pocket infection	Alive (under long-term suppression with daptomycin then linezolid)	> 870d
Radcliff [11], USA	1	75	Male	ICM	DT	1	28 m	Driveline infection (*Mycobacterium fortuitum*)	–	Early: cerebrovascular accident	Death (post-exchange day 7)	–
Alam [5], USA	1	61	Male	ICM	DT	2	3y	External compression of outflow graft from thrombotic, gelatinous materials	Redosternotomy + left subcostal approach	Early: sternal wound debridement	Alive	–
Ranard [14], USA	1	80	Male	ICM	DT	1	4y	AR and pump thrombosis	TAVR, then left subcostal thoracotomy	–	Alive	6 m
Our case, Taiwan	1	63	Male	DCM	BTT	1	1030d	Driveline damage	Limited left anterior thoracotomy + lower partial sternotomy	CMV pneumonitis, wound debridement and closure with local flap for previous wound of driveline insertion site	Alive	1y

*LVAD* left ventricular assist device; *ICM* ischemic cardiomyopathy; *DCM* dilated cardiomyopathy; *HM2* HeartMate II; *HM3* HeartMate III; *BTT* bridge to transplantation; *DT* destination therapy; *MSSA* methicillin-sensitive *Staphylococcus aureus*; *VRE* vancomycin-resistant *Enterococcus faecium*; *CMV* cytomegalovirus; *AR* aortic regurgitation; *TAVR* transcatheter aortic valve replacement

*Data included five patients undergoing pump exchange from HVAD to HM3

Duration of previous LVAD ranged from 30 to 3722 days. Pump thrombosis (*n* = 25) was the most frequent indication of LVAD exchange, followed by infection (*n* = 7). Redosternotomy was the most approach (*n* = 15). One patient underwent redosternotomy combined with left subcostal thoracotomy. Eight patients received thoracotomy, including lateral, posterolateral, anterolateral, or subcostal incision. Nine patients received left lateral thoracotomy extending toward a subcostal incision combined with a separate upper midline abdominal incision. One patient underwent limited left anterior thoracotomy and lower partial sternotomy. One study did not report the surgical procedure. One old HMII device was not removed, and the new HM3 outflow was connected to descending aorta.

Respiratory complications (*n* = 6), such as respiratory failure, prolonged intubation, and pneumonia, were the most common early complication after pump exchange. Other complications during the early postoperative period included dialysis, cerebrovascular accident, wound debridement, recurrent device thrombosis, and reoperation (one for pocket site bleeding, one for inflow malposition). During follow-up, one patient had a single syncope and several low flow alarms, and four patients had pump pocket infections (three with surgical drainage and debridement, one under long-term antibiotics suppression). Death or reoperation for another pump replacement was observed in four patients. Duration of follow-up ranged from 6 months to 3 years, except three studies did not provide relevant information.

## Discussion and conclusions

Previous reports have demonstrated that pump exchange from HMII to HM3 was feasible and safe; however, most clinical experiences were in Europe and the USA. There is a need for more information concerning Asian patients. To the best of our knowledge, this is the first case report presenting an Asian patient undergoing HMII pump exchange to HM3 successfully.

According to the literature review, nearly half of the patients received redosternotomy for HMII exchange to HM3. In our patient, we performed limited left anterior thoracotomy and lower partial sternotomy to avoid redosternotomy. Although the incision of pump exchange is mainly determined by the position of the pump and inflow graft, our procedure provides an alternative and limited approach to pump exchange.

Among the patients in the literature review, only our patient underwent HMII exchange to HM3 due to driveline damage. In our patient, the driveline damage possibly resulted from fatigue failure due to repetitive flexing and abrasion against the braided metal shield. We did not consider exchanging the HMII pump to HMII because of two reasons. The driveline design of the HM3 is better than the HMII, with a lower risk of driveline damage [15]. Additionally, it is hard for the patient to accept pump exchange to the same LVAD model, which did not work after only three years of use, instead of exchange to a new LVAD model.

This literature review showed that pump thrombosis was the most frequent indication of pump exchange, but only one (3.0%) patient encountered recurrent pump thrombosis after exchange. As the MOMENTUM 3 trial results showed that the HM3 group had a significantly lower risk of pump thrombosis than HMII [2], our review implied that the superiority of HM3 to HMII might not only be in primary implantation but also device exchange. However, this review also found four (12.1%) patients experiencing death or another pump replacement, comparable with the results of INTERMACS reports [1]. Therefore, much research is needed before a definitive conclusion can be drawn.

Infection is a common adverse event of LVAD, regardless of primary implantation or device exchange. The present review noted that infection was the second most common reason for pump exchange, consistent with the previous reports [1, 4]. Notably, four (12.1%) patients encountered pump pocket infections after surgery. HMII and HM3 have different characters in pump size and shape, location to place, outflow length, and insertion angle. With surrounding adhesive tissues, the old HMII pump pocket might become a dead space and a nidus of infection after pump exchange. Similar reasons could explain another case in the literature review, who received reoperation for inflow malposition on post-exchange day 7. Therefore, more attention should be paid to these potential complications after surgery for patients undergoing pump exchange to a different device.

In this case report, the patient successfully underwent pump exchange from HMII to HM3 and did not experience any hemodynamic adverse events or device malfunction during postoperative follow-ups of 12 months. The case demonstrated that it is safe and feasible to perform HMII LVAD exchange to HM3 through a limited thoracotomy and lower partial sternotomy for Asian patients.

## Supplementary Information

**Additional file 1.** Methods of the literature review.

## Abbreviations

CPB: Cardiopulmonary bypass
LVAD: Left ventricular assist device
LVEF: Left ventricular ejection fraction
HMII: HeartMate II
HM3: HeartMate 3
