# Supplementary material for: Left ventricular assist device exchange from HeartMate II to HeartMate 3 in an Asian patient—a case report and literature review
Source: J Cardiothorac Surg. 2023 Mar 7;18:82. doi: 10.1186/s13019-023-02133-4 (PMC9990556; doi:10.1186/s13019-023-02133-4)
Supplement: Supplementary file 1 — Additional file 1. Methods of the literature review. [file 13019_2023_2133_MOESM1_ESM.docx]

**SUPPLEMENTARY INFORMATION**

**Additional file 1**

**Methods of the literature review**

***Search strategy***

MEDLINE, Cochrane Library, EMBASE, and CINAHL Plus were searched until 31 January 2022. The following search terms were used: The following operators were used: ("ventricular assist device" OR "assist device" OR "assisted circulation" OR LVAD OR VAD) AND (exchange OR replace OR upgrade). The reference lists of the relevant studies were also searched manually to identify additional studies.

***Selection criteria***

The included studies were those that reported the patients undergoing LVAD exchange from HMII to HM3. When the same authors published multiple studies during a similar period, only the studies with the complete data were included due to the potential of overlapping patient data.

***Study selection and data extraction***

Two independent reviewers conducted literature searches based on the search strategy to identify eligible studies. A third reviewer would be if there were any uncertainty about study eligibility. The following information was extracted from eligible studies: the name of the ﬁrst author, year of publication, study design, demographic data, cardiology diagnosis, the indication of previous LVAD, duration of previous LVAD, the indication of pump exchange, surgical procedures, outcomes and complications, and duration of following-up.

After identifying 717 records, 579 were excluded, and 138 articles were left for full-text review. One hundred twenty-eight articles were excluded after reviewing the full-text articles, and the remaining ten studies were included for review.
